# Event-based feature tracking in a visual inertial odometry framework

**DOI:** 10.3389/frobt.2023.994488

**Published:** 2023-02-14

**Authors:** José Ribeiro-Gomes, José Gaspar, Alexandre Bernardino

**Affiliations:** Instituto Superior Técnico, University of Lisbon, Lisbon, Portugal

**Keywords:** event cameras, pose estimation, visual inertial odometry (VIO), unscented Kalman filter (UKF), Lie groups

## Abstract

**Introduction:** Event cameras report pixel-wise brightness changes at high temporal resolutions, allowing for high speed tracking of features in visual inertial odometry (VIO) estimation, but require a paradigm shift, as common practices from the past decades using conventional cameras, such as feature detection and tracking, do not translate directly. One method for feature detection and tracking is the Eventbased Kanade-Lucas-Tomasi tracker (EKLT), an hybrid approach that combines frames with events to provide a high speed tracking of features. Despite the high temporal resolution of the events, the local nature of the registration of features imposes conservative limits to the camera motion speed.

**Methods:** Our proposed approach expands on EKLT by relying on the concurrent use of the event-based feature tracker with a visual inertial odometry system performing pose estimation, leveraging frames, events and Inertial Measurement Unit (IMU) information to improve tracking. The problem of temporally combining high-rate IMU information with asynchronous event cameras is solved by means of an asynchronous probabilistic filter, in particular an Unscented Kalman Filter (UKF). The proposed method of feature tracking based on EKLT takes into account the state estimation of the pose estimator running in parallel and provides this information to the feature tracker, resulting in a synergy that can improve not only the feature tracking, but also the pose estimation. This approach can be seen as a feedback, where the state estimation of the filter is fed back into the tracker, which then produces visual information for the filter, creating a “closed loop”.

**Results:** The method is tested on rotational motions only, and comparisons between a conventional (not event-based) approach and the proposed approach are made, using synthetic and real datasets. Results support that the use of events for the task improve performance.

**Discussion:** To the best of our knowledge, this is the first work proposing the fusion of visual with inertial information using events cameras by means of an UKF, as well as the use of EKLT in the context of pose estimation. Furthermore, our closed loop approach proved to be an improvement over the base EKLT, resulting in better feature tracking and pose estimation. The inertial information, despite prone to drifting over time, allows keeping track of the features that would otherwise be lost. Then, feature tracking synergically helps estimating and minimizing the drift.

## 1 Introduction

Vision plays a very important role in the animal kingdom, and virtually every higher order animal has developed some sort of visual system to improve their chances of survival. As such, it is only natural that sensors that can imbue artificial systems with the sense of sight have been created, in particular cameras (what we call throughout this work as “conventional cameras,” referring to the familiar method of image acquisition, where frames are produced at a constant rate, to distinguish from event cameras, explained briefly). However, conventional cameras do not exactly mimic animal’s visual system. They are much slower (typically produce around 20–30 fps), produce redundant information, and are much more energy-costly. Furthermore, they are not very good with scenes with high contrast (as detail is lost in bright and dark areas), or with high movement (as images produced suffer from motion blur).

Neuromorphic hardware appears as a bio-inspired approach to hardware development that tries to replicate the advantages of animal systems, either in speed, energy efficiency, or any other positive or desirable attribute. Our work focuses on the use of the Dynamic Vision Sensors type of neuromorphic cameras [DVS cameras (Lichtsteiner et al. 2008)], which are a type of event cameras that report changes in the brightness captured by each pixel (precisely, the log-intensity of the brightness captured by each pixel). Unlike conventional cameras, that record a sequence of the intensity of all pixels in the scene, and therefore produce redundant information, and are not energy efficient, event cameras produce “events,” which contain the timestamp, pixel location, and polarity of the change in the pixel.

This approach has multiple advantages, such as 1) lower latency (because there is no need for video compression at the camera level), 2) higher energy efficiency, 3) no redundant information, 4) higher temporal resolution, in the order of microseconds, as opposed to milliseconds of conventional cameras, and 5) higher dynamic range, to name a few.

With said advantages, scenarios where speed is relevant are a natural candidate for the use of event cameras, and two such areas are that of pose estimation, and SLAM, which have used event cameras with success (Zihao Zhu et al., 2017; and Vidal et al., 2018). One keypoint in common with multiple approaches and methods is the need of feature detection and tracking, with the paradigm of event cameras in mind.

This work appears with the goal of improving on feature tracking using conventional cameras, by combining the feature tracker proposed in Gehrig et al. (2020) with the Unscented Kalman Filter based on Lie groups proposed in Brossard et al. (2017), in effect creating a synergy between the two systems.

## 2 Event cameras

Event cameras are image sensors that respond to changes in brightness in the scene. Unlike conventional cameras, which capture full image frames at a fixed frequency, commonly 30 Hz or 60 Hz, produce redundant information and require a high bandwidth for transmission, each pixel in an event-based camera operates independently and asynchronously, react to changes of brightness in the scene, eliminate the transmission of redundant information, allow for much higher temporal resolution, in the order of microseconds, as opposed to the milliseconds of conventional cameras.

Event cameras are inspired by the behaviour of the cells in the retina. Oversimplifying, retinal cells respond to changes in the environment (namely brightness), generating electrical impulses. The transient response of each retinal cell is independent. Event cameras mimic this behaviour by asynchronously and independently responding to changes in brightness in the environment, at the level of each pixel, generating ON/OFF events each time a predefined threshold in brightness is exceeded.

**FIGURE 1:**
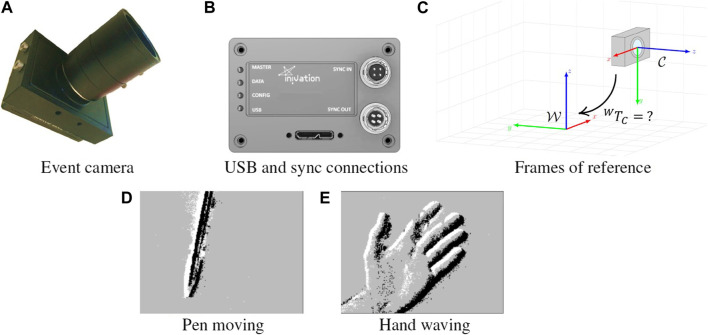
**(A)** *iniVation DVS240* event camera, **(B)** the available connectors, **(C)** relevant coordinate frames, namely world frame W and camera frame C . Outputs of event cameras, pen moving in front of the camera **(D)** and a person’s hand waving in front of the camera **(E)**. White and black denote the polarity of the event being represented, in particular positive and negative, respectively.

Events are triggered when the brightness in a certain pixel surpasses a certain threshold. In particular, discrete brightness steps are pre-defined, and whenever brightness detected crosses the threshold, an event is generated. Positive crossings generate ON events, and negative crossings generate OFF events. In effect, each pixel is constantly working as a comparator, with corresponding electronic to support this mode of working. Figure 1 shows example outputs of event cameras, when recording a pen moving, or a person waving.

This architecture allows for interesting properties, such as microsecond temporal resolution, high dynamic range (above 120 dB), which allows for scenes with both bright and dark zones, and does not suffer from under/overexposure, nor motion blur.

Events are then defined as a four-component vector

$$e={\left({\left(x,y\right)}^{T},t,pol\right)}^{T}={\left(p,t,pol\right)}^{T}.$$ (1)

The component $p={\left(x,y\right)}^{T}$ refers to the spatial position of the event in the camera. The component *t* refers to the timestamp of the event, and is of extreme importance due to the microsecond temporal resolution of the camera. Lastly, the parameter *pol* refers to the polarity of the event (ON/OFF events).

With this event structure, it is common to represent events in a three-dimensional (space-time), representation, where each event is a point with 2D spatial component, coupled with its 1D timestamp.

Conventional cameras and event cameras have fundamentally different modes of operation and output. As such, a comparison of the behaviour in the same scene, and an analysis of the output, is interesting. Let us analyse the response of both a conventional camera and an event camera when presented with a disk with a black dot rotating at a high speed. The fixed capture rate of the conventional camera is unable to keep up with the speed of the dot, and the images suffer from motion blur and some discontinuity between frames. The event camera, however, due to its asynchronous event generation and high temporal resolution, is able to continuously produce events relating to the movement of the dot.

Advances in camera manufacturing have allowed for cameras that have both conventional camera pixel arrays, and event camera pixel arrays. This enables hybrid algorithms, which take advantage of the benefits of event cameras, with the extensive research on conventional cameras.

## 3 Overview of related works

### 3.1 Event cameras, feedback loops, and pose estimation

Event cameras have demonstrated to be useful in multiple tasks where speed is paramount, of which quadrotor control comes immediately to mind (Mueggler et al., 2014). They have also been adopted in areas such as flow estimation (Akolkar et al. 2018), and image and video reconstruction (Rebecq et al. 2019), to name a few areas.

The main idea behind this work is that of a feedback loop, where the event-based feature tracking is improved by combining it with a pose estimator. This notion of combining two systems by means of feedback loop with the aim of improving the global performance is not novel, but not necessarily the most common. Works such as (Oberweger et al., 2015) use this idea in the context of the pose estimation of a hand, where the result from the main neural network is fed back into the system, by means of a secondary neural network.

(Chen et al., 2020) proposes a feedback loop in the form of a Madgwick filter in order to improve the pose estimation of a stereo VIO-system. The main takeaway is that such approach allows to have better estimates as there are multiple components that can benefit from the current state estimation.

More closely related to our work, Bai et al. (2019) proposes a feedback loop in the context of visual inertial odometry, where information from the Kalman filter used in the pose estimator is fed into the keyframe detector, in order to improve initial estimation, thus improving the pose estimator.

Our work borrowed from this idea, by merging the feature tracker proposed in Gehrig et al. (2020) with the pose estimator described in Brossard et al. (2017), and creating a feedback loop between the two, so that the feature tracker could benefit from the current pose estimation, leading to better features, in turn improving pose estimation.

The problem of pose estimation shares some goals and similarities with SLAM (Simultaneous Localization and Mapping), as one of the problems is that of localization, which relies on a correct estimation of the pose of the system. According to (Gallego et al., 2019), the first work on camera tracking with an event camera was presented in Weikersdorfer and Conradt. (2012), and proposed an implementation based on particle filters, but was limited to a planar motion.

(Reinbacher et al., 2017) proposed an implementations which, event though limited to rotation, and therefore without the need of translation or depth, paved the way for more complex implementations, such as (Vidal et al., 2018), which we consider to be the state of the art in terms of localization using event cameras.

Multiple authors have opted for approaches that try to rely on bridging the “classic” approaches with event cameras. For example, Zihao Zhu et al. (2017) relies on features tracked by Zhu et al. (2017), and combines them with IMU information by means of an Extended Kalman Filter (EKF). Our proposed approach borrows from this idea as well. Furthermore, the feedback loop contained in our work that we present as a contribution can be considered to expand on this work.

On the “conventional” side of pose estimation, multiple SLAM approaches based on vision (Visual SLAM, or vSLAM) are worth mentioning, however we highlight ORB-SLAM (Mur-Artal and Tardós 2017).

### 3.2 Feature detection and tracking with event cameras

For event-based cameras, new types of features, as well of detectors, are being proposed, as classical techniques are not easily transferable in most cases, or result in a non-negligible performance decrease, due to conversion overhead from asynchronous events to frames. Due to the nature of events, gradient operators are not possible (at least directly applied to the event stream), since there is no image on which to apply them, and multiple techniques have been proposed.

The work from (Clady et al., 2015) introduces the use of a space-time representation of events for feature detection and tracking. In this case, features correspond to image corners.

This method relies on the space-time properties of events, and creates a 3D representation, containing the spatial position of an event (*x*, *y*), as well as the time it was received. In this representation, edges moving with uniform linear speed create planes (stack of lines at different instants), and corner movement creates lines (stack of points at different instants).

As such, this technique tracks moving edges by fitting planes in this 3D representation, implicitly estimating the speed of the moving edge (optical flow). Each new event is matched to the previously estimated planes, and the estimates are updated. The way this technique identifies (and tracks corners) is by detecting intersection between these planes, as these intersections correspond to the corner movement through a period of time.

The approach proposed in Vasco et al. (2016) relies on the Surface of Active Events (SAE), a representation system for events, which keeps track of the timestamp of the most recent event for any given pixel, regardless of polarity, defined by *SAE*: (*x*, *y*) → *t*. Indeed, it is a spatial representation ((*x*, *y*) coordinates, corresponding to each pixel), which can be discretised by assigning a value to each pixel based on its timestamp. Since this discretised representation is now a frame in the classical sense, we can apply the Harris Corner Detector directly to the SAE and identify the corners from these results.

A more efficient implementation relies on considering only the neighbouring region of an event as it arrives, instead of the whole SAE. As such, only a subset of the SAE is analysed. Since each event, and consequently, each subset, is independent on the other subsets (provided the subsets do not overlap), parallel implementations are possible, and also improve speed. Such method is described in Mueggler et al. (2017), which merges the SAE with the FAST method of feature detection.

This technique also relies on the SAE representation of events but does not perform any computations. Rather, it performs only comparison operations on a local neighbourhood around the relevant event.

As each event is received, its timestamp is compared with the neighbouring pixels using circular segments (for isotropic response and efficiency), and checked if, in the region, each event is subsequently older than the central, current event (contiguous pixels with decreasing timestamps), as these are typical corner patterns.

Though this method is not as effective, it is much faster, as no computations are performed, and each event can be processed independently (and concurrently in a parallel fashion).

Lastly, the Event-based Kanade-Lucas-Tomasi Tracker, EKLT (Gehrig et al., 2020), is a hybrid feature tracking technique that is able to merge information from conventional cameras and events (and hence is more suitable for event cameras that output these two types of data simultaneously), that tracks corners across time. This method tracks corners, as they are easy to recognize in both conventional cameras and correspond to areas with high event generation.

The idea behind this tracker is to detect features using a conventional frame, which are then tracked using events until a new frame arrives, at which point the estimation from events is compared to the corner detection in the new frame, in essence correcting this estimation. If the feature is not detected, it is still tracked in event space, as subsequent frames may re-detect missed features. This approach is particularly useful in high-speed movements, where motion blur becomes a problem for frames, but not for events.

This comparison between frames and events is crucial, and the key concept is “image variation in a frame patch.” As previously discussed, event cameras respond to brightness changes in the environment. Therefore, it is not farfetched to compare events to image gradients, as zones with higher gradients in the world are precisely the ones that produce the most events. In fact, integration (accumulation) of events over a period of time produce results that are very similar to the gradient of the image.

This is the idea at the core of this approach, as the brightness change behaves as the descriptor for the features, and are used as patches for a Lucas-Kanade inspired patch comparison and matching, using both the patch and estimated velocity (estimated through events).

While a new frame is not received, the corner is tracked in event space and the local patch is being created for comparison with a frame patch created from image gradients.

### 3.3 Contributions

We propose a Lie group-based UKF approach to solve the pose estimation problem which, to the best of our knowledge, is a novel approach in the context of event cameras. This approach attempts to combine visual information in the form of events and frames, with inertial information obtained from an IMU, by means of an Unscented Kalman Filter.

Also, we propose the use of the Event-based Kanade-Lucas-Tomasi tracker (EKLT, Gehrig et al. (2020)) in the context of pose estimation, which, as far as we are aware, has not yet been done before.

Furthermore, we propose a possible way to improve said tracker by taking into account the current estimation of the pose, in effect improving sensor output by combining it locally (at the sensor level) with measurements from outside the sensor, which is an unusual approach to improving sensor reading, extending the idea present in works such as (Zihao Zhu et al., 2017).

## 4 Problem formulation

This work appears in the sequence of attempting to estimate the orientation of an eye by means of visual odometry. The eye produces very fast (under 200 ms) and short (usually under 25 deg) movements, called saccades, on the order of 700 deg/s (Luo, 2015). This poses some challenges for conventional cameras, that are susceptible to motion blur, and therefore lose features during this period. In order to minimize the effect of motion artifacts on feature tracking, exploratory work using event cameras was pursued. Given the availability of the embedded IMU sensor in most event cameras, inertial information was also used, which can be biologically justified by the synergy between the ocular (visual) and vestibular (inertial) systems in animals.

As such, our system is composed of an event camera that contains an IMU sensor embedded. It is important to understand what each sensor is reading and what reference frame each one uses to make sense of the data being fed into, and received from, the system.

Our IMU reports two types of information: angular velocity $\omega ={\left[\omega_{x}\omega_{y}\omega_{z}\right]}^{T}\in R^{3}$ and linear acceleration $a={\left[a_{x}a_{y}a_{z}\right]}^{T}\in R^{3}$ . There is no magnetometer information available. The IMU reference frame is aligned in the same way as the camera reference frame, and their centres are also aligned. As such, there is no need to change information between reference frames.

The setup is shown in Figure 1, showing the event camera with embedded IMU, the world frame W , with regards to which we want to estimate the position and orientation of our system (for the pose estimator), and the camera frame C , with regards to which the sensor readings are produced.

The odometry methods presented in this work intend to estimate the position of the camera, at all times, with regards to the initial (base) frame, typically the world frame W . Given the interest in estimating an eye orientation, focus was placed on rotation movements.

## 5 Feature tracking with event cameras

### 5.1 Feature detection and tracking

As stated, there are multiple choices for feature detection and tracking using event cameras. EKLT stands out as an hybrid approach that takes information from conventional frames and events. Being based on the Lucas-Kanade tracker, two templates need to be compared. The first is obtained by combining the *x*-wise and *y*-wise gradients along the estimated flow angle *v*. The second is obtained by temporal accumulation of the incoming events on a given patch. In effect, the first template tries to predict the generated events over the timestep, and the second template corresponds to the actual accumulation of events over that timestep.

These templates are then matched using the cost function

$$\underset{p,v}{\text{min}}{\left\|\left.\Delta L\left(u\right)-\Delta \hat{L}\left(u,p,v\right)\right)\right\|}^{2}$$ (2)

Where Δ*L* denotes changes from events (second template), $\Delta \hat{L}$ denotes gradients from frames (first template), and *u* denotes the image, *p* the warp parameters, in particular the position of the feature, and *v* the flow angle. *p* and *v* are used as the starting values for the optimizer that minimizes the functional (2), and are output at the end of the optimization process. The importance of these parameters is further explained in Section 7.2.

**FIGURE 2:**
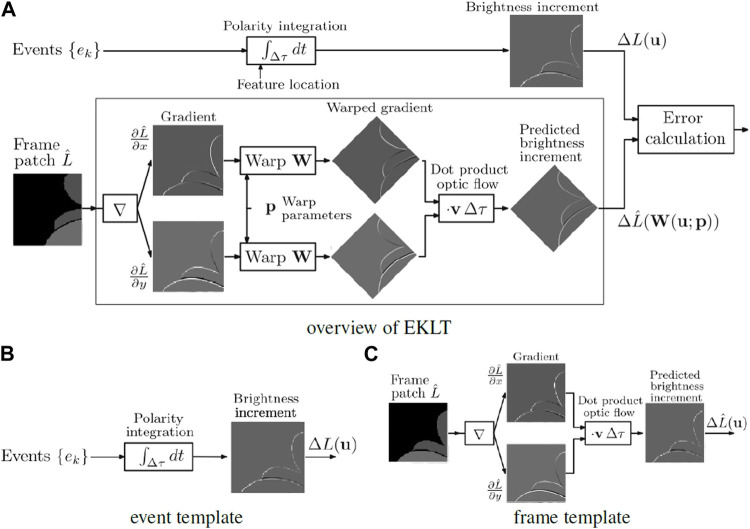
Block diagram of EKLT **(A)**, illustrating the comparison between brightness changes from images and events, adapted from (Gehrig et al., 2020). Comparison of the brightness change from event integration **(B)**, *versus* the brightness change from image gradient **(C)**.

The overview of the algorithm as described is shown in Figure 2. While a new frame is not received, the corner is tracked in event space and the local patch is being created for comparison with a frame patch created from image gradients, as shown.

It is worth noting, however, that the dependence on corners can present a problem for low-textured, or highly organic environments, where high quality corners are not always present. Also worth mentioning is the parameter *v* shown in Figure 2, which accounts for the optical flow. Though inconspicuous at first glance, *v* is crucial for the generation of the Predicted Brightness Increment, as it estimates the flow angle (the direction objects in the image are moving), which is needed to predict the polarity of the events and generate the template based on frames to compare against the real Brightness Increment generated from events.

This parameter is estimated by one of the following methods.

#### 5.1.1 Kanade-lucas-tomasi tracker (KLT) method

This approach uses the classic KLT algorithm (Lucas and Kanade, 1981) to estimate motion flow. The original algorithm estimates the motion flow by comparing patches between consecutive image frames (images *I* and *T* by means of the minimization of the photometric error

$$\underset{p}{\text{min}}{\left\|\left(I◦W\right)\left(u\right)-T\left(u\right)\right\|}^{2}$$ (3)

Where *W* (*u*; *p*) denotes a warp that maps image *I* to image *T*, and parameters *p* include the translation and rotation of these patches. From this warp, flow can be estimated.

We are already considering frame patches around features. This approach takes this into account and compares consecutive patches to estimate their motion, and estimate *v*.

#### 5.1.2 Event method

A second approach for optical flow estimation of the patches is based on the brightness constancy formulation (Zhang and Chanson, 2018), with a novel approach using events for the estimation of the derivatives with regard to time. However, an exact explanation is not needed for this work, and a detailed description can be found on the original work (Gehrig et al., 2020).

### 5.2 Event features and filtering

Since events do not naturally follow this organized and expectable pattern of producing visual information at a constant interval (which can be both advantageous and disadvantageous depending on the situation), changes are needed to provide a batch of features to the measurement model.

The proposed approach is that of accumulation of the asynchronous features over a period of time, in order to simulate frames being received. We call these accumulation of event features over time pseudo-frames, and are usually of 5 ms or less, to take advantage of the speed of events. Note these pseudo-frames only contain feature position on frame, and should not be confused with an event accumulation over a given period, much less an image reconstruction from events.

Three strategies are proposed for the creation of the pseudo-frames.

• Fixed interval integration Features are accumulated over a fixed period of time before being fed into the system. This period of time is defined at the beginning as is configurable by the user. Typical values were under 5 ms.
• Fixed number of features update Features are accumulated until a batch with a predetermined number of features is achieved.
• Hybrid approach Features are accumulated until a fixed period of time has passed, or until a predetermined batch of features is achieved (whichever comes first), and is a combination of both previous suggestions.

We found that a fixed interval integration was much more easily manageable as it translates quite naturally to a conventional camera producing features at a constant rate, albeit at a much faster rate. Therefore we used the fixed interval integration when validating the approach.

## 6 Pose estimator

In this section we explain our proposed approach for pose estimation using event cameras, leveraging both visual and inertial information provided by event cameras, based on the work from (Brossard et al., 2017) and the proposed FUSION pose estimator. This filter introduces several suggestions worth mentioning, such as 1) a Lie group structure for the state space (resulting in a matrix state space), 2) integration of the landmark position in the Lie group, and 3) representation and computation of the uncertainty directly in the Lie group (as opposed to outside of it, followed by a subsequent conversion to the Lie group).

### 6.1 System and measurements model

#### 6.1.1 State space

The state being estimated by the filter is given by the tuple $\left(\chi ,b\right)$ where *χ* is defined as

$$\chi =\left[\begin{array}{cccc} R & v & x & p_{1}\cdots p_{p}\\ 0_{\left(p+2\right)\times 3} & & I_{\left(p+2\right)\times \left(p+2\right)} \end{array}\right]$$ (4)

which incorporates the orientation **R** ∈ *SO*(3), velocity $v\in R^{3}$ and position $x\in R^{3}$ , as well as the 3D positions of the landmarks $p_{1},\ldots ,p_{p}\in R^{3}$ . The size of *χ* is (3 + 2 + *p*) × (3 + 2 + *p*). In addition, one has the bias vector $b\in R^{6}$ , defined as

$$b=\left[\begin{array}{c} b_{\omega }^{T}\;\;b_{a}^{T} \end{array}\right]$$ (5)

containing the gyroscope and accelerometer biases *b*_*ω*_ and *b*_*a*_, which is appended to the state, augmenting it.

#### 6.1.2 Dynamics model

The system can be modelled by

$$\text{body state }\left\{\begin{array}{cc} & \dot{R}=R{\left(\omega -b_{\omega }+n_{\omega }\right)}_{\times }\\ & \dot{v}=R\left(a-b_{a}+n_{a}\right)-g\\ & \dot{x}=v \end{array}\right.$$ (6)

$$\text{IMU biases }\left\{\begin{array}{cc} & \dot{b_{\omega }}=n_{b_{\omega }}\\ & \dot{b_{a}}=n_{b_{a}} \end{array}\right.$$ (7)

$$\text{landmarks }\dot{p_{i}}=0,\;i=1,\ldots ,p$$ (8)

where we have access to angular velocity *ω* and linear acceleration *a* through the IMU mounted on the system. *n* represents the various noise, defined as

$$n={\left[n_{\omega }^{T}\;n_{a}^{T}\;n_{b_{\omega }}^{T}\;n_{b_{a}}^{T}\right]}^{T}∼N\left(0,Q\right)$$ (9)

The notation (*ω*)_×_ represents the skew symmetric matrix associated with the cross product with vector $\omega \in R^{3}$ .

#### 6.1.3 Measurement model

Visual information is also fed into the system by means of a calibrated monocular event camera, in order to correct the predicted state of the system. The camera observes and tracks *p* landmarks through the standard pinhole model and corresponding projection model:

$$y_{i}=\left[\begin{array}{c} y_{u}^{i}\\ y_{v}^{i} \end{array}\right]+n_{y}^{i}$$ (10)

where *y*_*i*_ is the normalized pixel location of the landmark in the camera frames, and $n_{y}∼N\left(0,N\right)$ represents the pixel image noise.

This location is then compared with the expected location of the feature in camera space, obtained by projecting the estimated 3D position of the landmark into camera space through

$$\lambda \left[\begin{array}{c} x_{u}^{i}\\ y_{u}^{i}\\ 1 \end{array}\right]=\Pi \left[R_{C}^{T}\left(R^{T}\left(p_{i}-x\right)-x_{c}\right)\right]$$ (11)

where Π denotes our camera matrix, $R_{C}^{T}$ our initial rotation of the system, **R** ^*T*^ the current estimated rotation, **p**_*i*_ the *i* − *th* landmark 3D estimated position, *x* the estimated position of the system, and *x*_*c*_ the initial position of the system.

This visual information is provided by EKLT, as discussed in Section 5.

#### 6.1.4 Uncertainty on Lie groups

The usage of Lie groups to represent part of the state improves accuracy and numeral consistency, but comes at the cost of a more complex representation of noise. Since the state is not a vector space, the usual approach of additive noise is not possible. Following (Barfoot and Furgale, 2014), the probability distribution $\chi ∼N_{R}\left(\bar{\chi },P\right)$ is defined by mapping the uncertainty *ξ* to our state by means of the exponential map

$$\chi =\text{exp}\left(\xi \right)\bar{\chi },\chi ∼N\left(0,P\right).$$ (12)

The uncertainty $\xi ={\left[\begin{array}{c} \xi_{R}^{T}\xi_{v}^{T}\xi_{x}^{T}\xi_{p_{1}}^{T}\cdots \xi_{p_{p}}^{T} \end{array}\right]}^{T}$ is mapped to the Lie algebra through the transformation *ξ*↦*ξˆ* defined as

$$\xi \hat{\;}=\left[\begin{array}{c} {\left(\xi_{R}\right)}_{\times }\xi_{v}\xi_{x}\xi_{p_{1}}\cdots \xi_{p_{p}}\\ 0_{2+p\times 5+p} \end{array}\right].$$ (13)

#### 6.1.5 Time discretization

In order to implement Eqs 6–8, a simple discretization using the Euler method is used, with the exception of rotation. Considering a small time step Δ*t*, we have

$$\begin{array}{cc} R\left(t+\Delta t\right) & =R\left(t\right)\text{exp}{\left[\left(\omega \left(t\right)-b_{\omega }\left(t\right)\right)\Delta t+Cov{\left(n_{\omega }\right)}^{1/2}g\sqrt{\Delta t}\right]}_{\times }\\ v\left(t+\Delta t\right) & =v\left(t\right)+\left(R\left(t\right)\left(a\left(t\right)-b_{a}\left(t\right)\right)-g\right)\Delta t\\ x\left(t+\Delta t\right) & =x\left(t\right)+v\left(t\right)\Delta t\\ b_{\omega }\left(t+\Delta t\right) & =b_{\omega }\left(t\right)\\ b_{a}\left(t+\Delta t\right) & =b_{a}\left(t\right)\\ p_{i}\left(t+\Delta t\right) & =p_{i}\left(t\right) \end{array}$$ (14)

#### 6.1.6 Predict and update implementation

The various components of the system are described by

$$\text{state }\left\{\begin{array}{cc} & \chi_{n}=\text{exp}\left(\xi \right){\bar{\chi }}_{n}\\ & b_{n}={\bar{b}}_{n}+\tilde{b} \end{array},\left[\begin{array}{c} \xi \\ \tilde{b} \end{array}\right]∼N\left(0,P_{n}\right)\right.$$ (15)

$$\text{dynamics }\left\{\chi_{n},b_{n}=f\left(\chi_{n-1},u_{n}-b_{n-1},n_{n}\right)\right.$$ (16)

$$\text{observations }\left\{\begin{array}{c} Y_{n}={\left[\begin{array}{c} y_{1}^{T}\cdots y_{p}^{T} \end{array}\right]}^{T}≔Y\left(\chi_{n},w_{n}\right)\\ y_{i}\;\text{given by }\left(\text{11}\right),\;i=1,\ldots ,p \end{array}\right.$$ (17)

where $\left(\bar{\chi },{\bar{b}}_{n}\right)$ represents the mean estimate of the state at time *n*, $P_{n}\in R^{\left(15+3p\right)\times \left(15+3p\right)}$ is the covariance matrix that defines uncertainties $\left(\xi ,\tilde{b}\right)$ , and the vector **Y**_*n*_ contains the observations of the *p* landmarks with associated Gaussian noise $w_{n}∼N\left(0,W\right)$ .

These components are implemented into the filter, with the usual steps of propagation (based on the motion model with input from the accelerometer and the gyroscope) and update (based on the observation model and the visual information).

## 7 System overview and feedback loop

This chapter explains the approach to the pose estimation problem using event cameras, and (as designed) is only applicable to event cameras (though the idea can be converted to conventional cameras). It consists of creating a sort of “closed loop”^1^ between the filter, estimating the pose, and the tracker, tracking and providing visual features.

Two problems identified with a “clean” EKLT were as follows: the number of features is limited; and sometimes features are lost, only to be found a few moments later, but with a different ID (which is not necessarily bad, but then the filter treats this feature as a new one, and all previous sightings are discarded, which means some matching and filtering is needed). The first problem is of difficult resolution without major changes in the approach, as it is based on corner detection, which are common in images, but still limited. The second problem, however, implies improving the tracking of features so that they are kept alive for longer. As such, we set out to improve EKLT tracking performance.

Revisiting EKLT, at first glance it may seem like a simple implementation of KLT, where the matching patches are obtained from frames and events (as opposed to the normal strategy of both patches coming from frames), and, to a certain extent, this is true. But there is more to be said about the generation of these templates.

In Figure 2 we can see that the *x*-wise and *y*-wise image gradients are generated, and (after being subject to a warp) are merged by means of a dot product with the flow angle. This parameter of the flow angle *v* is critical in the generation of the template, and our experiments have verified empirically this parameter to be one of the main reasons for tracking loss. It can be interpreted as a weight in the linear combination of the *x*-wise and *y*-wise gradients of the image. If the camera is moving horizontally, then the accumulation of events is mainly on the horizontal direction, and *v* reflects this by placing more importance on the *x*-wise derivative. Movements in other directions have respective flow angle values that reflect this movement.

Furthermore, the flow angle *v* also allows to infer the direction of movement, as moving from left to right produces a different polarity of events to a movement from right to left. The template obtained from frames needs to take this into account to be able to be compared against the event template. Overall, this parameter is responsible for creating the global appearance of one of the templates to be matched. As such, it should now be clearer that this parameter is of paramount importance in the tracker.

Lastly, another parameter to be optimized is the initial location of the feature, which corresponds to the expected position of the feature, and is fed into the optimizer as a starting value. This parameter is also important, but since features are updated very frequently (sometimes around 1 ms, in very fast paced scenes) it is not as critical as the flow angle [though it also plays an important role, in particular when a feature is lost (Section 7.3)].

**FIGURE 3:**
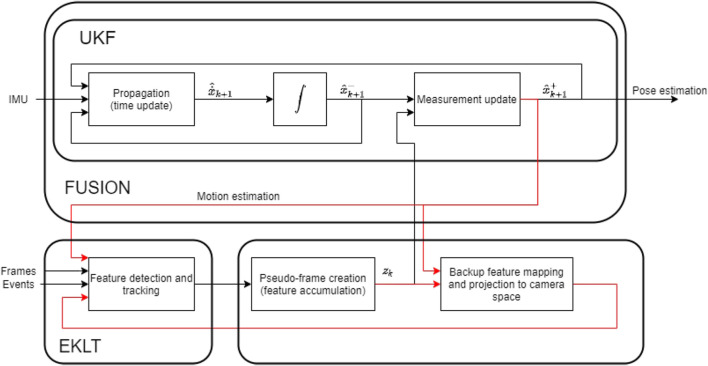
Overview of the proposed approach.

As such, parameter *p* guarantees that the patches are aligned, and parameter *v* guarantees there is a similarity between patches, hence its greater importance that we verified empirically. With this in mind, we propose an approach where the current estimated pose is fed back into EKLT to help with the tracking of features. This approach creates a sort of closed loop, where position from the pose estimator is fed into EKLT, which then provides information for the pose estimator, as shown in Figure 3. To the best of our knowledge, despite finding loop closing from motion to feature tracking in works as (Vidal et al., 2018 and Zihao Zhu et al., 2017), the loop closing is still an uncommon and innovative approach, specially when leveraging the filtered pose and optical flow prediction, together with combining with the EKLT.

This approach also tries to turn EKLT into a more robust alternative for feature tracking using event cameras that can only capture either frames or events at any given time, but can switch between these two modes, even though this switch may take some time. This is because, when features are inevitably lost, there is a period where no new information is provided, as no new frames are received for feature extraction, which reduces the contribution of the visual component. Therefore, by improving the tracking of features, this approach can also be beneficial to these cameras, as features are lost less, and their need to be replaced (and, therefore, of full frames), is reduced. Ideally, with perfect matching, only the initial frame would be needed. However, obviously, this is never the case, so a switch to frames (and then to events) is necessary to ensure tracking for these cameras.

### 7.1 Ego motion and optical flow

The movement of the features being captured by the camera are influenced by its motion (Heeger and Jepson, 1992), following

$$\left[\begin{array}{c} \dot{x}\\ \dot{y} \end{array}\right]=\frac{f}{Z}\left[\begin{array}{c} -T_{x}+\frac{x}{f}T_{z}\\ -T_{y}+\frac{y}{f}T_{z} \end{array}\right]+\left[\begin{array}{c} \omega_{x}\frac{xy}{f}-\omega_{y}\left(f+\frac{x^{2}}{f}\right)+\omega_{z}y\\ \omega_{x}\left(f+\frac{y^{2}}{f}\right)-\omega_{y}\frac{xy}{f}-\omega_{z}x \end{array}\right]$$ (18)

where $\dot{x}$ and $\dot{y}$ represent the flow in *x* and *y*-axes, respectively, *x* and *y* represent the feature in the image frame, *f* the focal length of the camera, *T*_(.)_ the translations of the camera, and *ω*_(.)_ the rotation of the camera.

Though such knowledge is obvious in the field of Computer Vision, given its relevance in this work, let us explore (18) in more detail. In particular, let us analyse the influence of each motion in the evolution of the movement of the features. The equation has been grouped in a translation component on the left, and a rotation component on the right. In total, there are 6 basic types of movement possible, corresponding to an isolated rotation or translation in the *x*, *y*, and *z*-axes.

**FIGURE 4:**
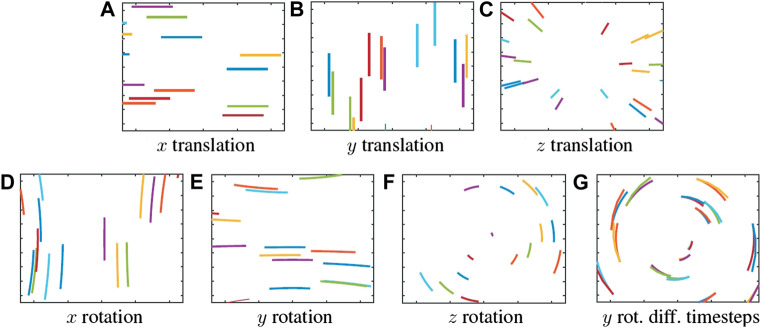
Evolution of features’ position over time due to ego-motion. **(A–C)** show translations in *x*, *y*, and *z*-axis, respectively, and **(D–F)** show rotations in *x*, *y*, and *z*-axis, respectively. Notice the similarities between **(A,E)**, and **(B,D)**. **(G)** shows drift in expected feature position from bigger timestep.

Exciting a single axis at a time produces the patterns presented in Figure 4, which show the position of each feature over time, accumulated on a single frame. Notice the similarities in pattern between rotation in the *y*-axis and translation in the *x*-axis, both of which produce mostly horizontal patterns, as well as rotation in the *x*-axis and translation in the *y*-axis, both of which produce mostly vertical patterns. These similarities also speak to the limitations of a purely visual odometry approach, as inertial measurement can help disambiguate between such motions. Only *z*-axis rotation and *z*-axis translation produce more distinctive patterns, the former producing concentric circles, and the latter producing lines moving into or away from the centre of the image.

Since we can predict the evolution of the position of the feature over time, it is possible to estimate the flow angle. However, it is very important to take into account that these predictions work for the immediate proximity of the feature, i.e., this assumption only works for small timestep. If the timestep is bigger, the predicted movement will not match the real position of the feature (see Figure 4, where features initiating at the same position eventually drift away and up at different positions).

### 7.2 Features tracking complemented by the pose filter state

Our objective is to improve the feature tracking from EKLT by feeding it information from the current pose estimation, which, in turn, will benefit from a greater number of features.

As already explained, the most critical (or, at least, the component that contributes most from loss of features) is the generation of the template to match from frames, which depends on two main components: the initial position location and the flow angle. Their importance (and relative importance) have already been mentioned (Section 5). We hypothesise the pose estimator can (either directly or indirectly) help the tracker with regards to these two components.

#### 7.2.1 Flow angle

Starting with the flow angle *v*, we propose the use of (18) to determine *v* by means of $v=atan2\left(\frac{\dot{y}}{\dot{x}}\right)$ where $\dot{x}$ and $\dot{y}$ are given by (18). We reiterate the importance of a small timestep, and synchronisation between the current estimate and EKLT, as disparities become more detrimental than beneficial. To tackle this problem, all timesteps are kept to a minimum, and are usually of about 1 ms. This ensures the assumptions for (18) are valid (according to our testing).

In terms of the estimations being used for motion, the angular velocity comes directly from the last measurement of the gyroscope (or some sort of mean or median of the last measurements, if readings are too contaminated by noise). The linear velocity, on the other hand, comes from the state, that estimates the filter velocity (along with system rotation and position, landmark position, and sensor bias). However, it is important to remember the relevant frames of reference (see Figure 1). The velocity being estimated is in the world frame W , meaning it needs to be first converted to the correct frame of reference (the camera’s) before being applied to (18). Luckily, since the rotation of the camera **R** is one of the variables being estimated in the state, the conversion of velocity to the camera frame is given by $v_{C}=R^{T}v_{W}$ where $v_{C}$ and $v_{W}$ denote the velocity *v* being referenced in the world frame W , and the camera frame C .

#### 7.2.2 Feature position

Moving on to the initial feature position, our proposed filter structure keeps the estimated 3D position of landmarks in the state, which can be projected into camera space to obtain the estimated position of the features by means of the projection equation

$$\lambda \left[\begin{array}{c} x_{u}^{i}\\ y_{u}^{i}\\ 1 \end{array}\right]=\Pi \left[R_{C}^{T}\left(R^{T}\left(p_{i}-x\right)-x_{c}\right)\right]$$ (19)

where Π denotes our camera matrix, $R_{C}^{T}$ our initial rotation of the system, **R** ^*T*^ the current estimated rotation, **p**_*i*_ the *i* − *th* landmark 3D estimated position, *x* the estimated position of the system, and *x*_*c*_ the initial position of the system.

This way, the tracker benefits from having an additional information of the features being tracked by adding the depth factor.

#### 7.2.3 Desired effect

In effect, by “helping” the tracker with the starting values, what is being done is placing the initial estimate inside the region of convergence, and closer to the global minimum, as the rest of the matching is still performed by the optimizer, that tries to match both templates, and estimate the current position of the feature (and its flow) in the process.

The analysis of this region would be interesting, i.e., to say that convergence is guaranteed whenever the initial flow angle is less than 5°, or when the initial position is not further than 3 pixels. However, such an analysis is not trivial, as there are many factors that come into play. To name a few, the speed and overall motion of the scene are critical and produce different scenarios.

A slowly moving, poorly distinctive feature produces less events (as the changes in brightness are fewer), and therefore take longer to create a patch, which means a greater displacement is produced, and without outside help of the closed loop the initial position is farther away from the minimum, perhaps outside the region of convergence. Not only this, but the patch that is created itself is usually not as distinctive, which result in suboptimal solutions to the flow estimation that result in suboptimal patches for comparison, and a poorer tracking of position overall.

From our experiments, the importance of the flow angle is much greater than the initial feature location. This is because the neighbourhood of the feature is really small (the displacement between initial location and final location is typically around 2-3 pixels diagonally), whereas flow angle could have deviated significantly from the last optimization (imagine a rotation in the *z*-axis of the camera, where features on the borders of the camera move faster than those on the inside), and influences the next matching negatively.

### 7.3 Set of backup features

The representation and management of features and landmarks is nothing new, and is a point where much effort is placed. Referring back to ORB-SLAM (Mur-Artal et al., 2015), for instance, the local map being generated is constantly being updated and corrected by means of local bundle adjustment, so that when new features are detected, they can be compared against the local map, which consists of a 3D point cloud (with additional parameters that simplify matching but are not particularly relevant in this case).

Based on this idea of using the map created over time to help with localisation, we try to take advantage of the location of features over time to help with localisation.

The implementation of the proposed closed loop approach also has other benefits. Tracking features in EKLT is costly (computation-wise). As such, when features are lost (either because their tracking quality decreasing under a certain threshold, or because they move outside the FOV of the camera), they are dropped.

In our case, the pose estimator, on the other hand, is capable of storing features and landmarks over time (in effect, creating a sort of map, as per a SLAM formulation) and keeping these lost and discarded features in a sort of zombie or dormant state.

Since we can project their predicted location onto camera space by means of (19), it is possible to awaken these features when they enter the FOV again, for example. This means that these features (that would eventually be detected again, but would be given a new ID, which would not benefit from using past sightings of these features), are able to be re-identified as used in the filter with the same ID.

This method also allows for bigger jumps in feature tracking, as the initial feature position can be set to a place that is far away from the previous estimated position (imagine a situation where this landmark becomes occluded, and therefore disappears, but is kept in memory by the pose estimator; when the landmark is no longer occluded, since the pose estimation kept running, the expected position based on current pose can be used).

Internally, this structure corresponds to a table that keeps track of all the sightings of a specific feature across time (based on its ID), in particular its *x* and *y* position in camera space, as well as the estimated camera pose (position and rotation) at that instant. Through the multiple points of view, associated with the feature position and camera pose, it is possible to triangulate the position of the landmark in 3D space, in the world frame (using epipolar geometry constraint).

Since the 3D position is being estimated, it is possible to project it into the camera frame at current time, and feed it into EKLT when it re-enters the camera FOV.

An interesting side effect of such an approach is that it is possible to rank features based on how distinct and/or observable they are, as features that are detected more, have more entries in the table, and therefore are probably the ones we want to use, as they are more robust.

### 7.4 Filter initialisation

The initialisation of the filter should be carefully considered. The “ideal” initialisation would be to provide the correct values of all variables in the state, i.e., set the correct values of pose and the location of the landmarks, in particular. However, not only is such an approach not always possible or realistic (testing on a new, uncontrolled environment for the first time), it also defeats the purpose of a system that is placed in an unknown environment and must be robust enough to eventually converge to the correct values.

This problem is not exclusive to our approach, and some suggestions have been made elsewhere. Taking (Qin et al., 2018), for example, a suggestion where a dataset is captured, and a first pass on the first moments of the dataset is performed, so that the first few values for the landmark location can be estimated (as multiple points of view allow for triangulation of features into 3D landmarks). After these values are obtained, the system starts again, initialised with them.

However, this solution only works offline, i.e., the system first captures a trajectory, and estimation is performed afterwards. Though perfectly legitimate, we preferred to strive for an approach where the system is able to run online, meaning it can estimate its location at the same time it is exploring the world, without the need for the first initialisation pass.

For the initialisation of the position, orientation, and velocity of the system, no prior information is given, and the filter starts with all values at 0. From our perspective, not only is this approach fair in the sense that the filter must be robust enough to be able to survive the first instants and quickly obtain these values, it also allows for quick testing in different environments, as no prior estimation is needed. Furthermore, this implementation assumes the starting pose to be the base frame, meaning all future pose estimations are given in relation to this frame, which we consider to be aligned with the world frame. If there are other components in the system, and we know this not to be true, a simple rigid motion transformation for a coordinate change can be performed. Also, if some information of the initial state of the filter is available, it can also be used for the initialisation, but it is not needed.

The landmarks, however, are a different story, and two distinctions for the initialisation are made: initialisation for the start of the system, and for the replacement of features. For the former, we place landmarks in the world by following the projection line of the corresponding features, which allows for estimation of the *X* and *Y* coordinates, but depth is trickier, as a single point of view is not enough for depth estimation (and for that matter, neither is rotation-only movement). In this regard, we assign a distance value *d* that reflects the average distance of the landmarks in the world, to have a notion of scale. As such, every landmark in the world follows |*X*, *Y*, *Z*| = *d* (which in practice means they are all in the same sphere of radius *d*). Another approach that was used for planar scenes was to consider the same *Z* for all features (they are all on the same plane at distance *Z* from the camera). Regardless, since the depth value is not trustworthy, a high variance is assigned to the start, which decreases as the system evolves.

For the latter (feature replacement), since information of past states of the filter are available (in particular, we keep the previous sightings of every feature), we can introduce a new landmark by triangulating the past sightings into the world, thus creating a much more reliable 3D position of the landmark that is introduced in the filter state.

### 7.5 Implementation

The block diagram of the implementation is shown in Figure 3. In this sketch, **x** represents the state, composed of the presented tuple (*χ*, *b*). $\hat{\left(x\right)}$ represents the estimate, ${\left(\hat{x}\right)}^{-}$ is the estimate before measurement update, and ${\left(\hat{x}\right)}^{+}$ represents the same estimate after update. *z* represents the observations (features) fed into the measurement update. Furthermore, the motion estimation that is being performed in the filter is being fed back into EKLT, and helps better keep track of the features by leveraging the predictable ego-motion effect on features, as well as the creation of backup features, that not only helps keep track of the features, but also re-identify them later.

We can consider there are two main components at work–EKLT for feature extraction, and the UKF for pose estimation–which work synergically. EKLT leverages the most recent pose estimation to obtain an estimate of the feature position using (19). Furthermore, the flow angle *v* is also estimated through (18) by using the angular velocity reading from the IMU that is fed into the UKF, and supplying that, or a filtered version of that reading, to EKLT. UKF, in turn, benefits from an improved quality of the feature being used as input. Algorithms 1 and Algorithms 2 reflect this explanation.

One possible comment is that *v* can be inferred from the derivative of *p*, which is true. However, given the low resolution of event cameras, the discretization error we get by computing the difference between consecutive positions may not be negligible. In addition, the errors in position contribute to an erroneous estimate of *v*. Furthermore, we estimate the whole movement of the system, and feed this information in the feedback, which is helpful to clear ambiguities due to the aperture problem. As such, both parameters are estimated separately.

In terms of computational cost, the closed loop is similar to the open loop implementation, as the computations of *p* and *v* are straightforward, see (19) and (18).

Algorithm 1 EKLT adapted for closed loop

**Require:** Event stream, pose estimation, landmark location, angular velocity
 Accumulate events
 **If** Enough events accumulated **then**
  Generate event patch
  **If** Frame received **then**
   Update frame patch
  **end if**
  Use (18) to obtain *v* initial estimate
  Use (19) to obtain *p* initial estimate
  Estimate feature position *p* using (2)
 **end if**
 **Output:** feature location

Algorithm 2 Pose estimator with closed loop

**Require:** Set of features (pseudo-frame), IMU values
 Initialize filter
 *χ*, *b* ← 0
 **while** there are IMU values **do**
  *χ*, *b* ← PropagationStep
  **If** Pseudo-frame received **then**
   *χ*, *b* ← UpdateStep
  **end if**
 **end while**
 **Output:** *χ*, *ω*, landmark location

## 8 Experiments and results

In order to validate the proposed approaches, simulation and real data was used. Simulated data was generated using ESIM ((Rebecq et al., 2018), an event camera simulator), and real data used datasets available online that use real event cameras ^2^, as well as recordings we performed using the Kinova robot arm to generate trajectories.

Given our group’s interest in the visual and vestibular system, the focus of this work, and therefore of the experiments presented, are directed towards rotational movements. Note that, despite our focus on estimating rotation, our methods allow estimating also translation. This is to account for real setups and datasets which do not perfectly realize rotations, i.e., rotation motions are contaminated with small translations.

### 8.1 Experiment 1, integrated experiment on simulation

To validate the approaches proposed, we generated a synthetic dataset based on ESIM, simulating a DAVIS240 event camera, with access to both frames and events simultaneously. The images produced have some distortion parameters, and some noise, but have no motion blur when simulating conventional frames, which would (in principle) give the upper hand to event cameras.

We generated simple rotations along each axis, with an amplitude of 18 deg for each side, inverting the direction of rotation when said angle was reached. This generated trajectory is simple, but the abrupt change in direction generated is a challenge for event cameras in general, as these correspond to instants with no events being produced, and EKLT in particular, as the estimated flow angle changes completely and instantly. Speed was 18 deg/s, so that the whole movement took about 4 s. A DAVIS240 camera was simulated, with both access to frames and events simultaneously.

**FIGURE 5:**
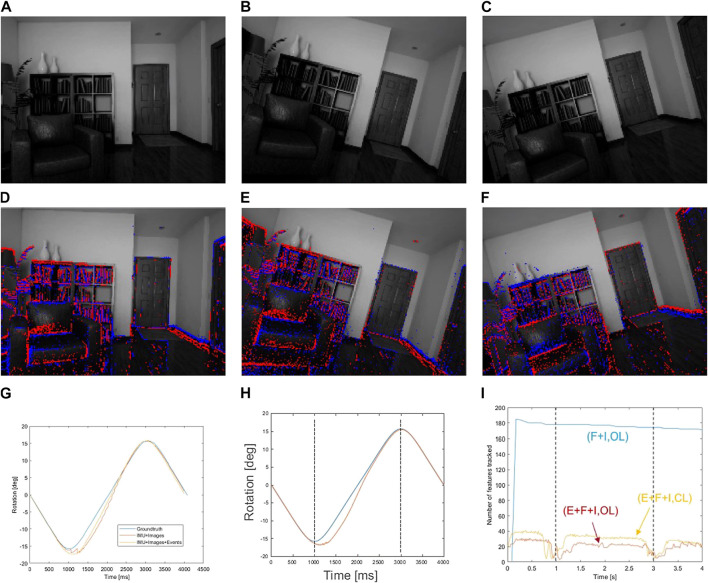
**(A–C)** show the evolution of the rotation along time, with rotation movement towards both sides on the *z*-axis. **(D–F)** show the same instants, but with the generated events superimposed. This input was generated using ESIM. **(G)** shows a comparison between the groundtruth (blue), the conventional approach (orange), and the event approach, open loop (yellow). **(H)** shows the comparison of the groundtruth (blue) with the closed loop approach (orange). **(I)** shows the evolution of the number of features for each approach used. Baseline (frames + IMU) is shown in blue. Event approaches, using events, frames, and IMU, are represented using red (open loop) and orange (closed loop).

The inputs to the system are shown in Figure 5, where we show 3 illustrative frames on the rotation movement, and superimpose the events on frames, making evident their relation with the edges and corners of objects in the image.

#### 8.1.1 Open loop approach

We start by the conventional approach, without using the feedback loop, as a baseline. In this case, we also performed a comparison with a conventional camera approach. The conventional camera approach is as described in the original proposed method in Brossard et al. (2017), and corresponds to a UKF with IMU, with visual features being extracted from a conventional camera. This comparison allows assessing whether event data can help improving VIO accuracy.

The results of this comparison are presented in Table 1. We observe that the approach with events outperforms the approach using frames, both in terms of the mean error, and the max error. This has to do with two factors: the more frequent updates that events allow mean that the filter can be updated with visual information more frequently; the features, being tracked with events, are tracked better (which can be debatable as it has their own problems, as lack of good descriptors, and being limited to corner features). We repeated this same setup, in the same scene, but with other motions, in particular rotations in the other axes. The results are summarised in Table 2.

**TABLE 1:** Results for conventional vs. event-based (open loop) approaches, when performing a rotation on the *z*-axis.

Setup	Mean error [deg]	Max error [deg]
Image + IMU	1.05	4.41
Image + Events + IMU	**0.85**	**3.05**

Bold corresponds to the best results.

**TABLE 2:** Open and closed loop results on simulation data.

	Open loop		Closed loop	
Axis of rotation	Mean error [deg]	Max error [deg]	Mean error [deg]	Max error [deg]
Rotation *x*-axis 18 deg	1.66	5.04	1.73	5.89
Rotation *y*-axis 18 deg	**0.65**	**2.43**	**0.049**	**0.12**
Rotation *z*-axis 18 deg	0.85	3.05	0.80	4.23

Bold corresponds to the best results.

Overall, the results validate the use of event cameras, even if with a simulator, and outperform the conventional camera approach in all cases (even though sometimes a change in the settings for the filter or the tracker is needed).

#### 8.1.2 Closed loop approach

After testing the first proposed approach (conventional approach without feedback loop), we present the result for the second proposed approach (feedback/closed loop) using the same scene and setup.

This approach adds some challenges, the most relevant of which is the need for a more careful choice of parameters for the filter. The estimation of the pose is now more closely coupled with the feature tracking. In other words, good pose estimations lead to better tracking, which leads to better estimation. A good starting estimation is critical to feature tracking.

The accuracies of pose estimation experiments are presented in Table  2. In the case of *x* and *y*-axis rotations, the former has biases that degrade the performance of the filter, resulting in a rotation that lesser accurate than the one estimated in open loop, however the latter performed much better. In a global view, these results corroborate that the closed loop approach does improve feature tracking, which, in turn, improves the estimation quality.

#### 8.1.3 Comparison of both approaches

The filter and IMU information is the same for all cases, which means that the differences in results are related to the visual features being fed into the filter. An evolution of the features over time is presented in Figure 5, where we can see the features for each case–traditional visual approach, open loop event-based approach, and the improved closed loop approach.

The first detail is the evolution of feature themselves, depending on the approach. The conventional camera approach is able to keep the number of features more or less constant, regardless of the time of movement being performed (though no motion blur was simulated). This is not the case with events, as they are dependent on movement for event generation and consequent feature tracking. Both event-based approaches have an evolution of features dependent on movement. In particular, there is a significant decrease of features at *t* = 1 s and at *t* = 3 s. These instants correspond to the change in direction on the movement, where flow angle changes instantly and no features are available for template creation.

The second detail worth mentioning is the number of features extracted from events is a magnitude of order lower than the features extracted and tracked from conventional frames. Nevertheless, this is not a problem, as the features tracked from events are more distinctive, and therefore more precisely placed in the world, and thus able for an overall better result on the tracking.

Furthermore, it is also worth comparing the number of features between open and closed loop approaches. The latter consistently tracks more features, corroborating the synergy proposed in the approach, which further improves performance.

Nevertheless, the closed loop approach helps keeping more features alive, for longer, as the number of features is consistently higher than the open loop approach. Furthermore, the recovery after the change is direction is also shorter.

Interestingly, results in *y*-axis closed loop in Table 2 are an order of magnitude lower then other cases. A combination of factors contributed to this result. In our dataset, the upper and lower part of the scenarios correspond to the ceiling and floor, which have a poor texture content. Thus, when the camera moves up or down, most of the image is occupied by regions with poor texture, which are lost frequently and must be re-identified. In particular, in the *x*-axis rotation, a total of 661 features were extracted from the scene, as opposed to 325 total features in the *y*-axis. This process results in a loss of accuracy.

Additionally, for the case of rotations along the *z*-axis, many features in the periphery go out of the field of view and thus have a lower lifespan. The most persistent features are located in the center, but have low signal to noise ratio. These problems are not so significant in the case of *y*-axis rotations and the accuracy is higher in general.

In the closed loop case, as the feature tracking is of overall higher quality, its effect on accuracy is even more noticeable, producing this particularly good result.

All in all, the proposed approach is able to better track features, keep them alive for longer, and recover faster after a major loss on the number of features.

### 8.2 Experiment 2, integrated experiment with a DAVIS camera dataset

To test the proposed approaches, we tested the performance on the datasets available online, which consist of a series of movements of the camera on multiple scenes. In particular, we tested the approach on the shapes and boxes datasets, as the former has clear contrast between background and shapes, and the latter has a much more textured environment.

#### 8.2.1 Open loop approach

We start with the first proposed approach. The results running this approach are presented in Table  3. We have decided to isolate each axis estimation for the sake of a less cluttered analysis, as well as to interpret the evolution of each axis independently. It is possible to observe that the obtained results are not satisfactory. First, there is an obvious drift in the *x*-axis that was not able to be compensated. We believe this drift is because of an uncompensated bias in the gyroscope, as this axis more easily loses features by moving out of the FOV, which means that the local map may itself drift overtime, and not correct sensor bias.

**TABLE 3:** Open loop on *shapes* scene.

Axes	Mean error [deg]	Max error [deg]	RMSE [deg]
*x*-axis	−30.49	−58.04	34.48
*y*-axis	−8.14	−45.70	17.35
*z*-axis	−7.03	−32.46	9.66

The results of running the open loop approach on the *boxes* scene are presented in Table  4. This experiment still produces some clear deviations on the true values. However, these results are slightly better than the ones presented previously, as not only are the errors smaller (with the exception of the *x*-axis rotation), as the overall profile of the estimation more closely follows the true values, which is positive.

**TABLE 4:** Open loop on *boxes* scene.

Axes	Mean error [deg]	Max error [deg]	RMSE [deg]
*x*-axis	−20.40	−50.10	30.88
*y*-axis	3.63	−10.25	6.13
*z*-axis	5.96	−12.27	8.80

#### 8.2.2 Closed loop approach

We tested the closed loop approach on the boxes scenario. The results running this approach are presented in Table  5. It is possible to see that the proposed approach does help with tracking, as the results show an improvement over the previous approach.

**TABLE 5:** Closed loop on *boxes* sscene.

Axes	Mean error [deg]	Max error [deg]	RMSE [deg]
*x*-axis	4.35	23.51	6.66
*y*-axis	−1.53	−10.77	5.11
*z*-axis	1.05	14.90	5.39

#### 8.2.3 Comparison of both approaches

Once again, it is interesting to analyse these results further and compare the open and closed loop appraoches. In particular, we choose to isolate the analysis of the *x*-axis, as this is the one with the highest error in both cases, and also the one where differences are more obvious.

**FIGURE 6:**
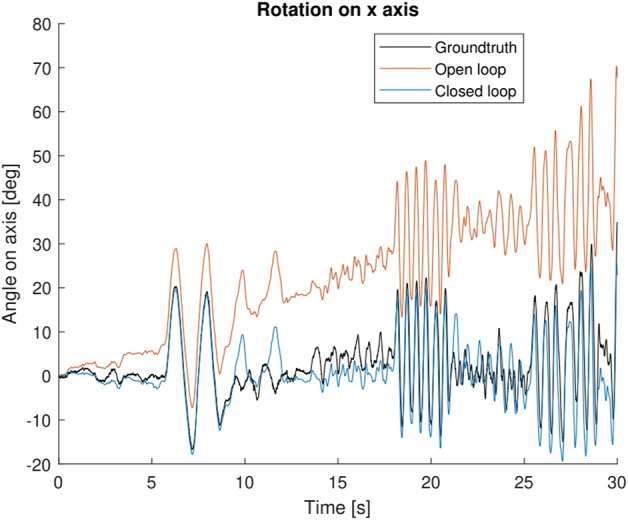
Comparison of the groundtruth (black) against the estimation of the open loop (orange) and closed loop (blue) approaches.

Figure 6 shows the plot of the estimation vs. groundtruth for both approaches. It is clear the bias from the IMU is not corrected by the visual component, and remains throughout the experiment. This is not the case for the closed loop approach, which is able to correctly estimate and cancel this bias, thus providing a much closer estimation to the groundtruth.

It should be noted, however, that the robustness of the closed loop is lower than the lower loop. The results are generally better, but the initial steps of estimating biases are critical, as an incorrect estimation can injure the tracker, because of the feedback from the state into the tracker, and the closed loop turns into a vicious cycle where estimation is not close to groundtruth.

### 8.3 Experiment 3, using the DVS camera mounted on the Kinova arm

**FIGURE 7:**
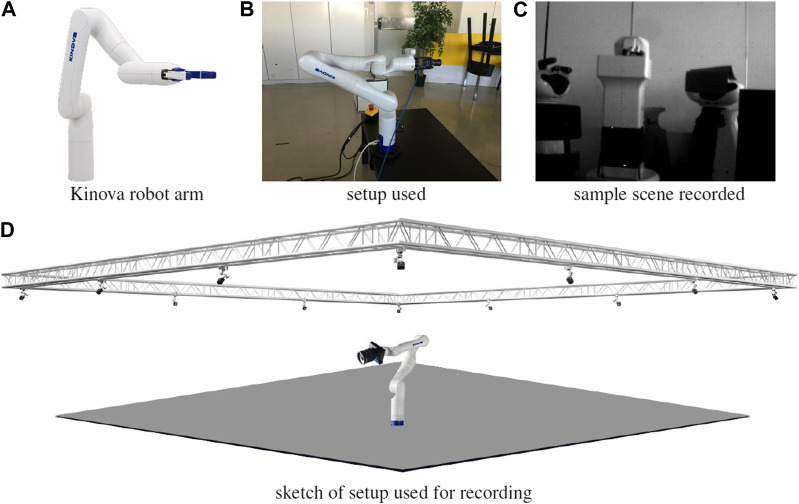
Setup for dataset recording in **(B,C)**, showing the Kinova robot arm **(A)** for trajectory generation and groundtruth recording, and surrounding motion capture system **(D)** for groundtruth recording. The DVS240 camera is coupled at the end of the arm, shown in **(B,D)**.

In this experiment, multiple rotation-focused movements were performed by means of a Kinova robot arm, with the hope of mimicking the eye saccadic movement, and being able to track it along time. This mimicking was mostly in terms of the velocity and acceleration profiles, not necessarily what is humanly possible (we consider torsional movements, which do not occur in the eyes, for example). The setup used for this experiment is shown in Figure 7.

Since the DVS camera is the camera considered for this experiment, either frames or events may be recorded at each time (exclusive or). The data recording encompasses two parts i) image frames when the camera is still, and ii) events when the camera is moving. The commutation from frames to events is not automatic, is placed in the script of the data acquisition. IMU is always recording.

**FIGURE 8:**
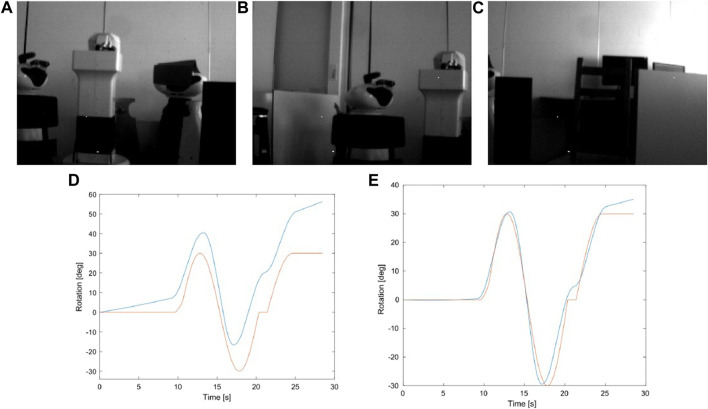
**(A–C)** show samples from the scene recorded for the experimnent using the Kinova robot arm. **(D)** and **(E)** compare the groundtruth (orange) with the estimation (blue) from the case where no prior is given **(D)** and when some information from the biases is used for initialization **(E)**.

After feeding this recording into EKLT it was verified that frames based features are effectively tracked, however the event based features are lost between tens to hundreds of milliseconds after detection, resulting in a drift in estimation. In a second experiment, we have calibrated the IMU and initialized the pose estimation method with estimated biases of the IMU. Under these conditions, though not perfect, the estimated rotation much closely follows the real value. Figure 8 shows the evolution of the estimation for these two cases.

In a third experiment, we took an hybrid approach leveraging the start of the recording, where the camera stays static until around 10 s, and therefore IMU output is mostly noise (and gravity). As such, we start by running the filter considering frames, as if we were using a conventional setup, in order to estimate bias, obtaining a RMSE of 0.3635 deg on the axis of movement, which is actually quite promising, though results mostly from a good estimation of the biases from the initial estimation from frames.

## 9 Conclusion and future work

In this work we developed a system for pose estimation based on event cameras. Two approaches were developed to tackle this problem. A first, which combined an Unscented Kalman Filter developed around a Lie group structure, with a feature detector and tracker based around events, which performed well under simulated environments, but ultimately under-performed in the real system. The second approach has shown promising results on simulation. In real environments, the initialisation of the filter is critical, as poor estimations lead to poor tracking. However, when the initialisation is carefully performed, the filter performed more accurately than using the first (open loop) approach.

Both proposed methods introduced new concepts that can be further improved. Interesting results were obtained and serve as a basis to understand the current status of event cameras, their limitations and advantages.

As future work, following machine learning approaches for pose estimation may be interesting. Depending on the type of data being used, for example if direct events are to be used, work on Spiking Neural Networks (SNN) may be used, as the asynchronous nature of events is best captured by the asynchronous nature of SNN.

## Data Availability

The raw data supporting the conclusion of this article will be made available by the authors, without undue reservation.

## Footnotes

1 Not to be confused with loop closure, as is common in SLAM formulations, but more like a control theory approach of open vs closed loop control.

2
http://rpg.ifi.uzh.ch/davis_data.html
